# Crystal Structures and Binding Dynamics of Odorant-Binding Protein 3 from two aphid species *Megoura viciae* and *Nasonovia ribisnigri*

**DOI:** 10.1038/srep24739

**Published:** 2016-04-22

**Authors:** Tom Northey, Herbert Venthur, Filomena De Biasio, Francois-Xavier Chauviac, Ambrose Cole, Karlos Antonio Lisboa Ribeiro, Gerarda Grossi, Patrizia Falabella, Linda M. Field, Nicholas H. Keep, Jing-Jiang Zhou

**Affiliations:** 1 Crystallography, Institute for Structural and Molecular Biology, Department of Biological Sciences, Birkbeck, University of London, London WC1E 7HX, UK; 2 Research Department of Structural and Molecular Biology, University College London, Gower St., London WC1E 6BT, UK; 3 Department of Biological Chemistry and Crop Protection, Rothamsted Research, Harpenden, AL5 2JQ, UK; 4 Laboratorio de Química Ecológica, Departamento de Ciencias Químicas y Recursos Naturales, Universidad de La Frontera, Temuco, Chile; 5 Dipartimento di Scienze, Università della Basilicata, via dell’Ateneo Lucano 10, 85100 Potenza, Italy

## Abstract

Aphids use chemical cues to locate hosts and find mates. The vetch aphid *Megoura viciae* feeds exclusively on the Fabaceae, whereas the currant-lettuce aphid *Nasonovia ribisnigri* alternates hosts between the Grossulariaceae and Asteraceae. Both species use alarm pheromones to warn of dangers. For *N. ribisnigri* this pheromone is a single component (*E*)-β-farnesene but *M. viciae* uses a mixture of (*E*)-β-farnesene, (−)-α-pinene, β-pinene, and limonene. Odorant-binding proteins (OBP) are believed to capture and transport such semiochemicals to their receptors. Here, we report the first aphid OBP crystal structures and examine their molecular interactions with the alarm pheromone components. Our study reveals some unique structural features: 1) the lack of an internal ligand binding site; 2) a striking groove in the surface of the proteins as a putative binding site; 3) the N-terminus rather than the C-terminus occupies the site closing off the conventional OBP pocket. The results from fluorescent binding assays, molecular docking and dynamics demonstrate that OBP3 from *M. viciae* can bind to all four alarm pheromone components and the differential ligand binding between these very similar OBP3s from the two aphid species is determined mainly by the direct π-π interactions between ligands and the aromatic residues of OBP3s in the binding pocket.

The accurate detection of semiochemicals (naturally-occurring behaviour/development-modifying chemical signals) in a surrounding complex environment is crucial for insects to survive, and a sensitive and specifically-tuned olfactory system is essential. In most insects, there is a highly selective and sensitive olfactory recognition system for individual volatile semiochemicals that are perceived at critical stages of the insect’s life cycle. The antenna is the main olfactory organ, where two olfactory protein families, the odorant binding proteins (OBPs) and the odorant receptors (ORs), are responsible for receiving, transporting and triggering responses to semiochemicals. OBPs, including the pheromone-binding proteins (PBPs), are small, globular polypeptides of 150–250 amino acids and have been described in a great number of insect species from most orders. The characteristic signature of most OBPs is the presence of six highly-conserved cysteine residues, with a conserved spacing, paired in three interlocked disulphide bridges, although atypical OBPs with more than six cysteine residues have been reported^1^.

Aphids (Hemiptera: Aphididae) use semiochemicals, such as plant volatiles and pheromones, to locate their host plants, find mates and warn of danger. There is good evidence that aphids, like other insects, use OBPs to transport semiochemicals across the lymph into the sensilla on their antennae and deliver the signals to the ORs, where a behavioural response is initiated. This includes the detection of alarm pheromones. Research on the pea aphid, *Acyrthosiphon pisum,* has shown that the recognition of the alarm pheromone (*E*)-β-farnesene (Eβf), and structurally related molecules, is mediated by ApisOBP3 and ApisOBP7^2^,^3^,^4^. Homologous genes to *ApisOBP3* have also been identified in other aphid species^5^,^6^ and in other insects such as the hoverfly, *Episyrphus balteatus* (EbalOBP3)^3^ and the brown plant hopper, *Nilaparvata lugens* (NlugOBP3)^7^ with these OBPs showing an apparent high affinity for Eβf. Indeed it was suggested that *E. balteatus* uses Eβf as a kairomone to locate its aphid prey^8^. The aphid OBP3s have been immunochemically localised to the aphid antennal sensilla^9^,^10^, as well as in non-sensory organs^10^, and RNA interference of NlugOBP3 has shown that it is involved in both nymph olfaction and more general non-olfactory functions^7^.

The vetch aphid *Megoura viciae* (Buckton) feeds exclusively on members of the Leguminosae, whereas the currant-lettuce aphid *Nasonovia ribisnigri* alternates its hosts between members of the Grossulariaceae and Asteraceae. When attacked, both species release an alarm signal to warn neighbouring aphids of danger. For *N. ribisnigri*, like *A. pisum* and the peach-potato aphid *Myzus persicae,* this signal is a single compound Eβf^11^,^12^,^13^,^14^,^15^,^16^. However, for *M. viciae* the alarm signal is a mixture of several terpenes [(−)-α-pinene, β-pinene, Eβf and limonene], although behavioural assays have shown that (−)-α-pinene is the most active component^13^,^14^,^15^.

The aim of the present study was to identify and characterise OBP3 from *M. viciae* and *N. ribisnigri* by molecular, biochemical and structural approaches and to determine whether this OBP is involved in detection of aphid alarm pheromone components. For the first time we report the crystal structures of an aphid OBP and postulate the molecular interactions with Eβf using affinity measurements and *in silico* binding prediction by molecular docking and molecular dynamics analysis.

## Results and Discussion

### Identification of OBP3 in *M. viciae* and *N. ribisnigri*

To identify genes encoding OBP3 in *M. viciae* and *N. ribisnigri*, which do not have genome sequences available, we used primers designed to match the ApisOBP3 mature protein (GenBank accession: FM242529), a strategy likely to work because of the high similarity between OBPs of different aphid species^6^. This allowed the successful cloning of the genes encoding mature MvicOBP3 and NribOBP3 (GenBank accessions: KT750882 and KR139668), which like ApisOBP3, can be classified as “classic” OBPs, with the usual conserved spacing between the six conversed cysteines^2^. The mature proteins have 118 and 114 amino acids with theoretical pIs of 5.20 and 5.18, and molecular weights of 13.7 and 13.4 kDa, respectively. In contrast to the high diversity amongst OBPs from the same species^6^, the OBP3 sequences are very similar between aphid species with ApisOBP3 and MvicOBP3 having only three amino acid differences, and NribOBP3 having two amino acid differences plus four fewer residues at the C-terminus (Supplementary Figure 1). There is one amino acid difference between MvicOBP3 and NribOBP3 plus four fewer amino acids at the C-terminus. Since ApisOBP3^2^,^17^ and the *Sitobion avenae* OBP3^3^ have been shown to be involved in the binding of Eβf, we hypothesised that MvicOBP3 and NribOBP3 might also serve as a carrier for Eβf. However, since *M. viciae* also has other terpene components in its alarm pheromone blend; it might be that its OBP3 can also bind these compounds. We therefore decided to determine the interactions of MvicOBP3 and NribOBP3 with Eβf, (−)-α-pinene, β-pinene and (+)-limonene as well as the crystal structures of these proteins.

### Expression and purification of MvicOBP3 and NribOBP3

We expressed MvicOBP3 and NribOBP3 in BL21(DE3) pLysS *E. coli* cells transformed with pET17b vectors containing the coding sequences for the mature proteins. The recombinant proteins, as is the case for most insect OBPs, were obtained as inclusion bodies and were therefore denatured with urea/DTT, refolded using the cysteine-cystine redox pair to enable disulphide bond formation and dialysed to obtain a soluble form. This process has been reported to give OBPs with native folding and with the correct cysteine bridges^18^,^19^,^20^,^21^. However, since it is also known that the expressed OBPs may contain endogenous ligands from the bacterial cells, they were delipidated prior to use^22^,^23^. The refolded OBPs were purified by anion-exchange chromatography followed by gel filtration, giving a single band by gel electrophoresis (Supplementary Figure 2).

### X-ray structure of MvicOBP3 and NribOBP3

Experimentally-determined 3D structures of eighteen different classic and two plus-C insect OBPs are available in the Protein Data Bank (http://www.rcsb.org/pdb/home/home.do) and many of these have different structures dependent on their crystal form, pH or ligand. There are a further 8 salivary, haemolymph or venom protein structures that share the Interpro domain motif (IPR006170) with OBPs, but differ in length and other structural features. In total there are 104 PDB files of IPR006170 proteins containing 171 chains (August 2015 search).

The OBP structures are from Diptera (mosquitos and fruitflies), Hymenoptra (honey bee), Lepidoptera (moths), Orthoptera (locust) and Dictyoptera (cockroach), but to date there are no such structures for aphid OBPs. Indeed there are no reports of an OBP 3D structure for any Hemipteran, which comprise around 50,000–80,000 species of aphids, planthoppers, leafhoppers, shield bugs, and other crop pests despite there being more than 300 Hemipteran OBPs reported in NCBI GenBank. Thus, we present the first 3D structures of OBPs from aphids, *M. viciae* (MvicOBP3) (PDB: 4Z39) and *N. ribisnigri* (NribOBP3) (PDB: 4Z45), by X-ray diffraction (Supplementary Table 1).

**Figure 1:**
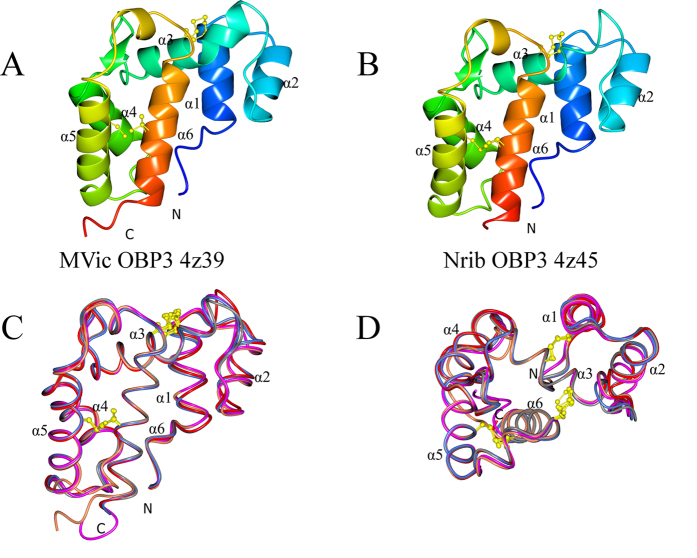
Cartoons of MvicOBP3 (top left) and NribOBP3 (top right) coloured from blue to red by residue number. The superposition of the 5 chains in these structure [MvicOBP3 (**A**) coral and (**B**) magenta, NribOBP3 (**A**) blue, (**B**) grey and (**D**) red] are shown in the same orientation (bottom left) and viewed from the top of the left view (bottom right) showing the variation is in helix 1 and 2 and the loop between helix 2 and 3.

The OBP3s from both aphid species have a similar structure to other classical OBPs i.e. six α-helices connected by extended loops, with three disulphide bridges formed by the six conserved cysteine residues between Cys17-Cys44; Cys40-Cys101 and Cys86-Cys110 (Fig. 1). The 5 chains in the two structures are reasonably similar (Calpha RMSD less than 1.0 Å) (Table 1, Fig. 1) with the variation being mainly in the loops and the first two helices.

**Figure 2:**
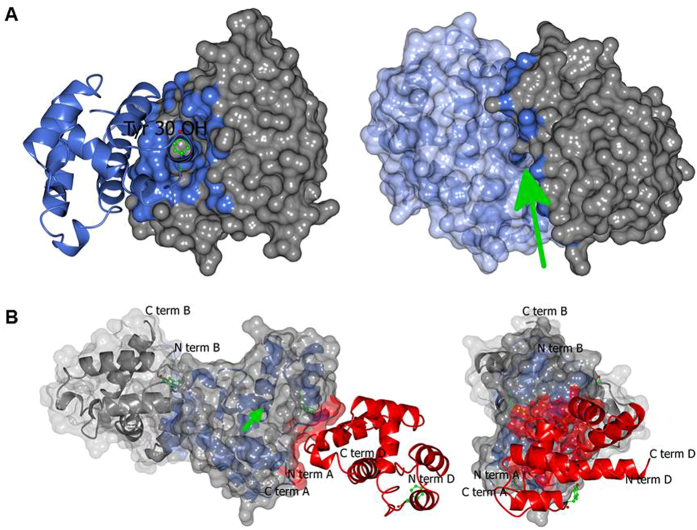
The protein-protein interface in the crystal structure of MvicOBP3 (A) and NribOBP3 (B). The green arrows indicate the binding pockets. Tyr30 is shown in green. (**A**) Left: the interface of chain A (grey surface with interface residues in blue) and chain B (blue ribbon) with TyrB30 (green). Right: the view of horizontal 90 degree rotation around the pocket entrance. Chain B is in a light blue surface. (**B**) Left: chains B (grey), A (blue) and D (red) in the filament. The surfaces of A and B are grey with the interface coloured by the interacting chain. Right: the end view of three fold rotation along the filament. The three green Tyr30s indicate the three fold.

The two OBPs also have some unique structural features which are different from other classical OBPs. There is a significant groove in the surface of the aphid OBP3s (indicated by an arrow in Fig. 2) formed primarily from the N-terminal region of the chains and parts of helices α1 and α2. In the two molecules of the asymmetric unit of MvicOBP3, each chain partially occupies the other’s groove, with Tyr30 inserted deep into the groove, forming an inter-chain hydrogen bond with the N of Phe5 in the other chain. The N-terminal residues of the other chain cover the second face of the groove and these symmetrical groove interactions form the centre of the monomer-monomer interface. The two molecules are related by very close to a twofold rotation of 179.4 degrees. A search of Protein Interfaces, Surfaces and Assemblies (PISA) (http://www.ebi.ac.uk/pdbe/prot_int/pistart.html)^24^ was used to analyse this interface, giving a calculated interface surface area of 1110 Å^2^ per chain. PISA predicts that an interface of this size is biologically relevant rather than being a purely crystal packing interface. The formation of the monomer-monomer interface has the observed solvation free energy gain (ΔiG) of -6.5 kcal/mol with the ΔGdiss of 13.5 kcal/mol and a p value of 0.463. The interface generates a pocket, which assessed by CASTp^25^ has a volume of 390 Å^3^. The volume of this pocket is bigger than the internal cavity of the *Antheraea polyphemus* PBP ApolPBP without ligand which has a volume of 282 Å^3 ^26 and that of *Bombyx mori* PBP BmorPBP1 which has a volume of 167 Å^3 ^27. However, it is smaller than the 492 Å^3^ of *A. mellifera* OBP AmelASP1^28^ and the 435 Å^3^ of *Leucophaea maderae* OBP LmadPBP^29^.

Protein-protein interactions of OBPs has been reported for some mosquito OBPs, such as CquiOBP1, AgamOBP1 and AaegOBP1, where the OBPs form a hydrophobic tunnel, which is tuned to bind long molecules, such as (5*R*,6*S*)-6-acetoxy-5-hexadecanolide, the mosquito oviposition pheromone (MOP), which binds to CquiOBP1. Each CquiOBP1 binds a single molecule of MOP, but the aliphatic chains of the two molecules come close at the dimer interface, and make some contacts with the second chain. There is a fenestration to the binding pocket near the head group of MOP in each molecule so the dimer face need not open up to allow binding^30^. In the very similar (90% identity) AgamOBP1 dimer, DEET molecules make a similar level of contacts as MOP across the dimer interface, with polyethylene glycol (PEG) molecules occupying the side entrance to the pocket^31^. A single larger, but non-physiological PEG binds to both subunits in AaegOBP1 and AgamOBP1 in the absence of other ligands^32^,^33^. The *A. mellifera* OBP AmelASP1 has a dimer formation that leads to domain swapping^28^. The interfaces of these OBPs bury 1200–1400 Å^2^ of the proteins so they are not much larger than that of MvicOBP3. Also the plus-C OBP, OBP48 from *A. gambiae*^34^, forms a predicted dimer burying an area of 2200 Å^2^ from each chain generating a large pocket with a volume of 4330 Å^3^ containing a PEG molecule.

**Figure 3:**
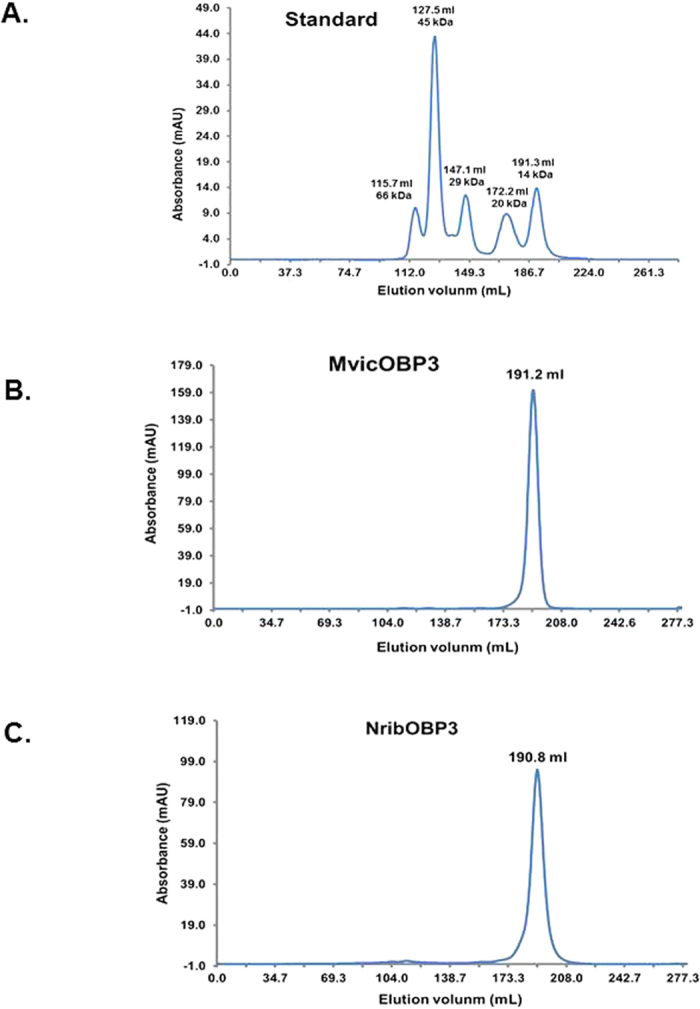
Gel filtration trace of protein standards, MvicOBP3 and NribOBP3. The protein elution volume is labelled above each peak. The protein standards are lactalbumin (14 kDa), trypsin inhibitor (20 kDa), carbonic anhydrase (29 kDa), ovalbumin (45 kDa) and BSA (66 kDa).

However, the interface seen in MvicOBP3 is not recapitulated with NribOBP3 (Fig. 2B). Instead the three chains in NribOBP3 are in a filament generated by an approximate three fold screw axis perpendicular to the crystallographic three-fold screw (Angles 117.9, 122.4 and 119.8 degrees and translations of 28.9–29.3 Å summing to the a and b axis length). This contact involves some of the same residues as the symmetric putative protein interface contact in MvicOBP3 (Arg4, Phe5, Thr7 and Tyr30), but buries a much smaller area (average 608 Å^2^) and would not form a closed dimer assembly. This combined with gel filtration evidence that both MvicOBP3 and NribOBP3 are monomers in solution (Fig. 3), indicates that the proteins are likely to function as monomers. We did not observe any time dependent dimerisation of MvicOBP3 as reported for some other OBPs^34^,^35^.

### Comparison of MvicOBP3 and NribOBP3 with other OBPs

To compare the aphid OBP3s with other OBPs in the PDB, searches on PDBefold and DALI were run with chain A of the two OBP3 structures. Using PDBefold MvicOBP3 gave 87 hits. Only the lowest hit by Z-score (PDB: 1M63) does not match IPR006170, this is also the 72^nd^ by Z-score and only non-match to IPR006170 of the 75 matches to NribOBP3. RMSD Calpha for these hits varies from 2.1–3.7 Å, number of aligned residues from 71 to 104 (out of 118 or 121) and sequence identity from 8–20% (Supplementary Table 2). A DALI server search with MvicOBP3 and NribOBP3 gave 167 and 160 hits respectively, before the first non-IPR006170 protein, with all 160 common to both hit lists. The RMSD of these fits ranges from 2.7 to 5.1 Å, with the number of aligned residues ranging from 80 to 111 and the sequence identity from 7–20%.

These analyses confirm that the sequence identity with other OBPs is quite low, and the RMSD with OBP structures is quite high. Not only is sequence diversity high in the OBPs, but there is also considerable conformational flexibility as agreements between different structures of the same OBP are often above 1.0 Å RMSD. The sequence alignment of the PDB representatives of the OBP family shows almost no conservation outside the conserved cysteines. This means it is not feasible to analyse the evolutionary conservation across the OBP family. The correlation between the DALI and PDBefold lists is only moderate. The Pearson Rank correlation coefficient when ordered on Z-score is 0.46 between the PDBefold and the DALI order for MvicOBP3 and only 0.23 for NribOBP3. Changing the factor used to rank the hits also shows only a moderate correlation, indicating that the two aphid OBP3s are related to other OBPs but there is not a subset of existing structures that matches well to the two aphid OBP3s by this automated comparison.

**Figure 4:**
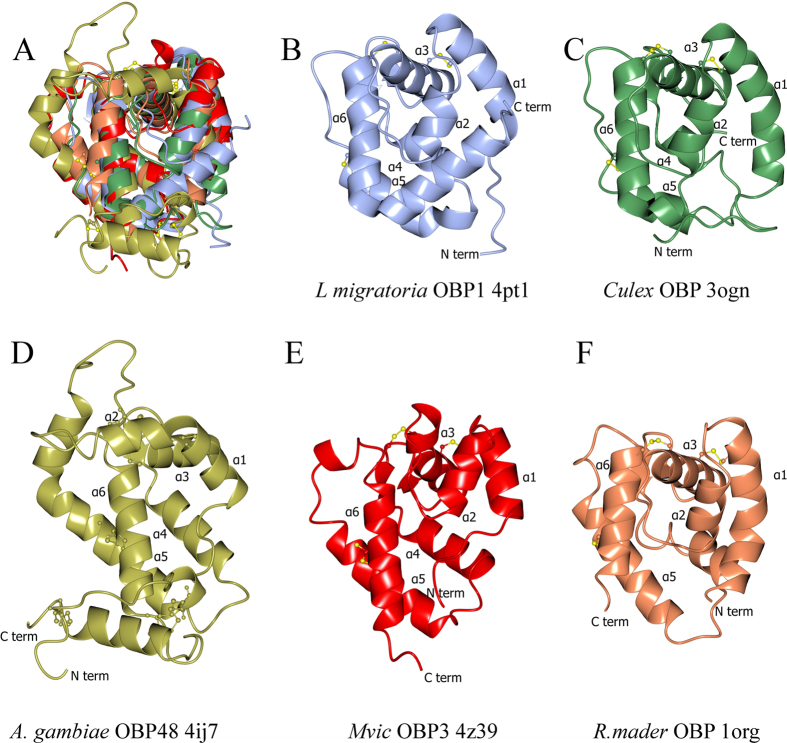
(**A**) Superposition of five OBPs matching panels B-F in the same orientation. (**B**) *Locusta migratoria* OBP1 (PDB 4pt1)^55^. This is a highly ranked structural homologue but has the conventional C-terminus, in this case a helix, coming across the bottom of the molecule to the right hand side as drawn; (**C**) *Culex quinquefasciatus* OBP (PDB 3ogn)^30^ has the C-terminus going across the bottom in a similar position to *L. migatoria*; although its N-terminus does come across to the left hand side; (**D**) *Anopholes gambiae* OBP48 structure (PDB 4ij7)^34^ has the C-terminus not coming across the bottom of the molecule and staying on the left as drawn, with the N-terminal coming across the bottom of the molecule as does (**E**) MvicOBP3 (PDB 4z39) and (**F**) *Rhyparobia mader* OBP (PDB 1org), although in this case the N-terminus does not block off the pocket fully.

Manual inspection shows that when compared to the vast majority of OBP structures the N terminus of the aphid OBP3 is in an unusual place close to the centre of the molecule, where the C terminus is normally found. Indeed only in the structures of the cockroach OBP (PDB 1ORG, 1OW4 and 1P28)^29^ and the longer plus-C atypical OBPs (PDB 4IJ7, 4KYN, 3PM2)^34^,^36^ does the N terminus head in a similar direction (Fig. 4).

No significant internal binding pockets were predicted by CASTp^37^ or SURFNET^38^ for both MvicOBP3 and NribOBP3. This absence of an internal binding pocket is in contrast to other OBPs that are known to bind a ligand in an internal cavity^26^,^27^,^28^. Molecular dynamic (MD) analysis over 10 nsec simulation showed that the structural conformation changes are minor (Supplementary Figures 3 and 4), indicating that there is probably not some cryptic central pocket that was somehow hidden by crystallisation.

### Ligand binding studies with MvicOBP3 and NribOBP3

**Figure 5:**
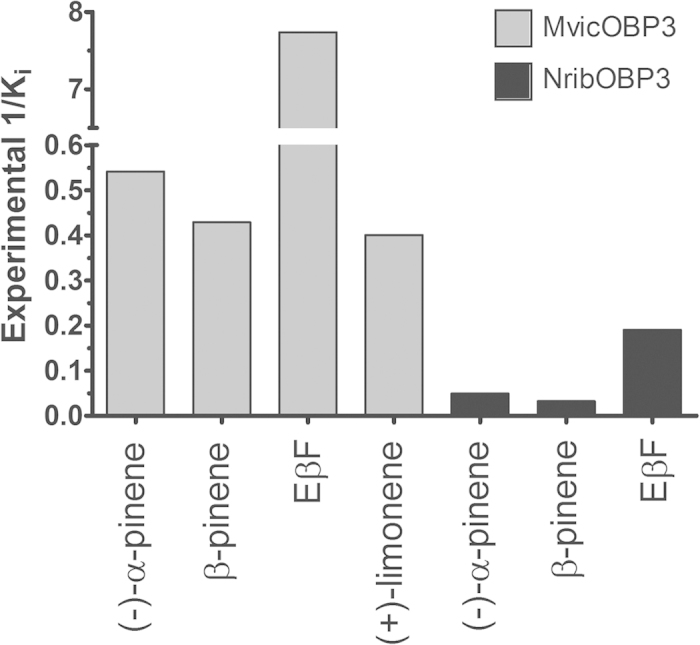
Competitive inhibitor binding constants (Ki) of MvicOBP3 and NribOBP3 to the aphid alarm pheromone components measured experimentally by the fluorescence displacement binding assay. For a more clear presentation, 1/Ki values have been plotted.

The unique structural properties (i.e. no internal binding pocket, positioning of N-terminus) prompted us to carry out ligand binding studies. Recombinant MvicOBP3 and NribOBP3 bound the fluorescent probe, N-phenyl-1-naphthylamine (NPN), with high affinity (K_d_ 1.9 μM and 9.0 μM, respectively) (Supplementary Figure 5), which allows other ligands to be tested in a competitive binding assay. Figure 5 reports the comparative inhibitor binding constants (K_i_) of four potential ligands, Eβf, β-pinene, (−)-α-pinene and (+)-limonene as determined by the displacement of NPN from MvicOBP3/NPN and NribOBP3/NPN complexes by increasing amounts of these chemicals. This shows that MvicOBP3 has a much higher affinity for Eβf (K_i_ 0.1 μM) than for the other alarm pheromone components β-pinene (K_i_ 2.3 μM), (−)-α-pinene (K_i_ 1.8 μM) and (+)-limonene (K_i_ 2.5 μM). NribOBP3 has a K_i_ value of 5.2 μM for Eβf, 20.2 μM for (−)-α-pinene and 30.7 μM for β-pinene. Therefore, MvicOBP3 and NribOBP3 could be involved in detecting and recognising Eβf, which is consistent with previous reports of other aphid OBP3s^2^,^3^,^4^. MvicOBP3 seems to have a stronger binding affinity to these semiochemicals than NribOBP3 in the fluorescent competitive binding assay relative to the binding of NPN (Supplementary Figure 5).

### Molecular docking

**Figure 6:**
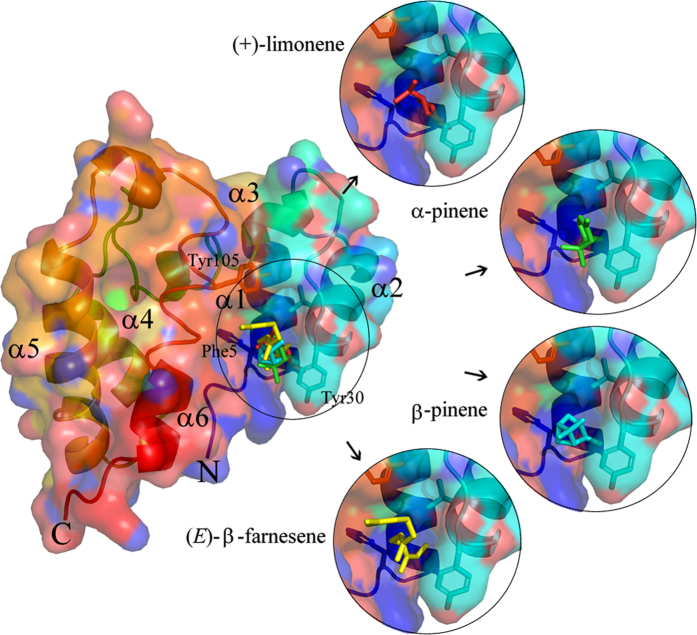
Superimposed binding modes of (−)-α-pinene (green), β-pinene (light blue), (*E*)-β-farnesene (yellow) and (+)-limonene (red) in the interface of MvicOBP3 predicted by blind docking.

A blind molecular docking approach was used with Eβf, β-pinene, (−)-α-pinene and (+)-limonene using Autogrid in Autodock Tools software with a grid box big enough to cover the whole chain A protein area of MvicOBP3 and NribOBP3. This indicated that the four ligands were mainly docked in the region of Tyr30 and in the groove. To further investigate the ligand binding, directed-molecular docking was performed on the chain A of MvicOBP3 using Autodock Vina, which is more precise and faster than its predecessor Autodock 4.0^39^. The best conformation for the lowest free binding energy for Eβf, β-pinene, (−)-α-pinene and (+)-limonene are given in Table 2 and shown in Fig. 6. The docking results indicate that there is no major difference in the binding energy of MvicOBP3 to (−)-α-pinene (−5.1 kcal/mol), β-pinene (−5.2 kcal/mol), (+)-limonene (−5.4 kcal/mol) and Eβf (−5.5 kcal/mol). The competitive binding assay results give the order of strength of binding of Eβf ≫ (−)-α-pinene > β-pinene = (+)-limonene (Fig. 5). The stronger binding of Eβf is not due to the formation of hydrogen bonds because there is a lack of functional groups such as alcohols, aldehydes or carbonyl groups in the ligands tested. The participation of residues Phe5, Val27, Tyr30 and Tyr105 in the docked conformations suggests that hydrophobic interactions are involved in the binding because of the hydrophobic nature of both the ligands and the protein residues. Some π-π interactions could also be present between Eβf and MvicOBP3 involving Phe5 and Tyr105, due to the presence of unsaturated bonds in Eβf, which are not present in the pinenes and limonene. There are differences in the binding affinities obtained between the fluorescent binding assay and the molecular docking, probably because molecular docking does not consider electronic properties, where the main structural differences of the two pinenes lies.

**Figure 7:**
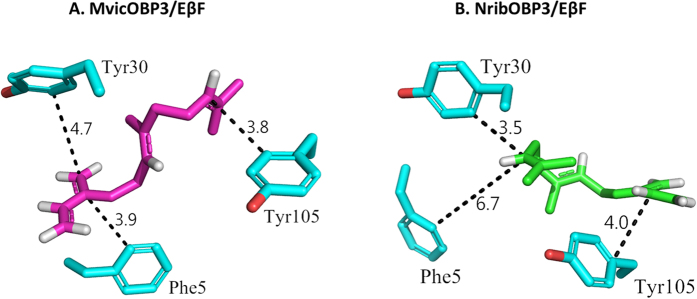
Docked conformation of (*E*)-β-farnesene (sticks) into the putative binding pocket on chain A of MvicOBP3 and NribOBP3. Hydrogens (white) in the ligand adjacent to unsaturated bonds are shown. Distance of the amino acid residues Tyr30, Phe5 and Tyr105 to (*E*)-β-farnesene in the binding pocket of MvicOBP3 (**A**) and NribOBP3 (**B**) is shown in Å and as black dashed lines.

The molecular docking gives a marginally weaker NribOBP3/Eβf complex than MvicOBP3/Eβf complex (−5.5 kcal/mol for MvicOBP3/Eβf and −5.1 kcal/mol for NribOBP3/Eβf) and subsequent K_i_ (92 and 181 μM, respectively) (Table 2). This is also in agreement with the experimentally determined binding affinity, where MvicOBP3 has higher affinity (K_i_ of 0.1 μM) than NribOBP3 (K_i_ of 5.2 μM) (Fig. 5). In MvicOBP3/Eβf complex, the double bonds of Eβf have a more direct interaction with the aromatic rings of Phe5 and Tyr105 (π-π interactions) in the binding pocket. Moreover, Eβf has more contacts with residues, such as Val27, Met108, Phe5, Lys28 and Tyr105. On the other hand, for NribOBP3/Eβf, the Eβf interacts more weakly with Tyr30, Arg4, Tyr105 and Ala109 with fewer contacts and has only one direct interaction with the aromatic ring of Tyr105. Tyr30 is inserted deep into the groove (Fig. 2 and Supplementary Figures 3 and 4) and quite close to the double bonds of Eβf (3.5 Å) (Fig. 7) but molecular docking suggests that its aromatic ring is not interacting directly with the double bonds as is in MvicOBP3/Eβf. This is supported by the C-like curved arrangement of Eβf in the NribOBP3/Eβf docked pose and the fact that the Phe5, an important residue in MvicOBP3/Eβf docked pose, is quite far away from the Eβf in NribOBP3/Eβf (6.7 Å) (Fig. 7). Docking studies of ligands to the MvicOBP3 dimer show that the best ligand/MvicOBP3 conformations are very similar to monomer MvicOBP3 complexes; all ligands are docked into the same surface binding site as in monomer MvicOBP3 although not as deep as with the monomer but with higher binding energy. This indicates that the binding pocket is the likely binding site.

Overall the docking studies indicate that the difference in the ligand binding between MvicOBP3 and NribOBP3 is probably not due to the last four amino acid residues at the C-terminus, (which is the major sequence difference), as they are on the opposite end of the protein to the ligand binding site as is the point mutation at Ile98 in MvicOBP3 and Val98 in NribOBP3 (Fig. 6 and Supplementary Figure 3). Another main structural difference between the OBPs is in the loop between helix 2 and 3 (Fig. 1), but the docking results indicate that in both OBPs all close interactions between ligands and protein are with amino acids from helices 6 and 2. This suggests that sequence difference is almost certainly not hiding a real difference in the ligand binding energy between these two very similar OBP3s.

The MD results of MvicOBP3 showed that Tyr30 is displaced towards the protein (Supplementary Figures 3 and 4). In contrast, the MD analysis for NribOBP3 shows that Tyr30 is not significantly displaced. The conformational energy of MvicOBP3 monomer is higher than that of NribOBP3 (722 kcal/mol vs 677 kcal/mol, repectively) indicating that it is not in a conformation as stable as NribOBP3 conformation. The most likely explanation is that Tyr30 is involved in the crystal packing/dimerisation in MvicOBP3 and is relaxing towards its probable monomeric conformation more like that seen in NribOBP3. It is also possible that these differences between the OBP structures are because of the different crystallisation pHs (NribOBP3 has been crystallised at pH 5.0 whereas MvicOBP3 at pH 7.5). However as Tyr30 lies close to the predicted ligand binding site, its flexibility may well also be involved in ligand binding either directly or via alterations to the shape of the binding site. We cannot rule out that the optimum binding site is in some other conformation of Tyr30. The conformational energy for the MvicOBP3 conformation point mutated to the NribOBP3 sequence is 710 kcal/mol which is 12 kcal/mol less than MvicOBP3 (722 kcal/mol). That implies that a conformational energy of 33 kcal/mol of the total difference in energy of 44 kcal/mole between NribOBP3 (677 kcal/mol) and MvicOBP3 (722 kcal/mol) could be ascribed to the movement of Tyr30 between the two conformations.

## Conclusions

Two OBP3s from the aphids *M. viciae* and *N. ribisnigri* were characterised by molecular, biochemical and structural approaches. For *M. viciae* OBP3 the fluorescence competition binding assays and molecular docking demonstrate that it binds strongly to Eβf, as seen for the *A. pisum* OBP3^2^,^3^,^4^,^5^. However, it also binds to β-pinene, (−)-α-pinene and (+)-limonene so this one OBP could be responsible for transporting all four components of the alarm pheromone blend of this aphid. Behavioural experiments have shown that (−)-α-pinene is the most active compound in producing the alarm response of *M. viciae*^40^,^41^ but in the present study both the fluorescence competitive binding assay and the molecular docking failed to show any real differential binding between (−)-α-pinene and β-pinene. In contrast NribOBP3 binds to Eβf with a lower affinity than does MvicOBP3 and has even lower affinity to the pinenes, which is consistent with the fact that *N. ribisnigri* does not use pinenes as its alarm signal.

The observation that most aphids studied so far just use Eβf as their alarm signal suggests that this is the ‘ancestral’ state and that *M. viciae*’s use of four components is relatively new. It would be interesting to see if *M. viciae* has other OBPs involved in discrimination of the alarm pheromone components. To this end there are 11 full length OBPs annotated in the *A. pisum* genome^6^ and homologous genes have been identified in *M. viciae*^42^.

The differences in the inhibitor binding constants (Ki) for the aphid OBP3s between the experimental values (Fig. 5) and the theoretical values (by molecular docking) (Table 2) could be due to the nature of the methods used in this study. In the competitive fluorescence binding experiments the inhibitor binding constants (K_i_) are measured indirectly through the displacement of the fluorescent probe and expressed as ligand concentration. Whereas, the theoretical ligand binding affinities are predicted by molecular docking (i.e. force field calculations based on molecular mechanics) with an empty binding pocket and expressed as free binding energies. Inhibitor binding constants are calculated from the free binding energies and expressed as K_i_ by the docking software. Moreover, the ligands in this study are considered as flexible, allowing torsional rotations^43^. Another factor that may contribute is the dynamic nature of the ligand binding, during which protein conformation may change, whereas the protein was rigid in the docking^44^.

MvicOBP3 and NribOBP3 are almost identical, and neither the sequence differences nor their C-terminus are contributing factors to their different 3D structure and ligand binding affinity. Molecular dynamic analysis demonstrates the residue Tyr30 (its interactions with ligand and orientation before and after ligand binding) may play an important role in the ligand binding. MvicOBP3 is in an open conformation (Tyr30 away from the pocket) because of the interaction with another monomer. The MD indicates that the MvicOBP3 would prefer to have Tyr30 in the NribOBP3 position (towards the binding pocket) in its monomeric state. The *in silico* binding studies may not have fully captured its role as calculated binding to the ‘closed’ Tyr30 in NribOBP3 and ‘open’ Tyr30 in MvicOBP3 structures are more or less the same.

Aphids are the main insect pests of agricultural crops in temperate regions, often causing major economic losses. Although broad-spectrum insecticides are available for control, alternative and more targeted methods are needed for sustainable agriculture due to insecticide resistance, environmental pollution and reduced availability of insecticides. One alternative method for aphid control is to exploit their pheromone-mediated communication^45^. We appreciate that the unique features of the two aphid OBP3s (no internal binding pocket, surface binding site and involvement of N-terminus in forming the binding pocket) needs further evaluation of the effects of these features on their biological functions and role in the aphids’ responses to alarm pheromones. In the longer term this could contribute to alternative methods for aphid control, by exploiting their pheromone-mediated communication for the interruption of their reproduction and for the attraction of natural enemy parasitoids and predators.

## Methods

### Insect rearing

*M. viciae* was reared on broad bean plants (*Vicia faba* L.) in pots in a chamber at 20 ± 1 °C, 75 ± 5% relative humidity (RH), under a light:dark 18:6 h photoperiod. The cultures were started from a clone originally collected from alfalfa plants in Southern Italy (Eboli, SA). *N. ribisnigri* was reared on lettuce (*Lactuca sativa*) in pots in a chamber at 23 ± 1 °C during the day and 19 ± 1 °C during the night with 60% RH and light:dark 14:10 h photoperiod.

### RNA extraction and cDNA synthesis

Apterous adults (50 mg) of *M. viciae* and *N. ribisnigri* were homogenised separately in 1 mL TRIzol RNA extraction reagent (Sigma-Aldrich, St. Louis, MO) and total RNAs isolated following the manufacturer’s protocol. The integrity and purity of the total RNA were determined by agarose gel electrophoresis and the concentration measured using a spectrophotometer (NanoDrop ND-1000). Before cDNA synthesis, the RNA samples were treated with amplification grade DNase I (Life Technologies, Carlsbad, CA), according to the manufacturer’s instructions. cDNA was prepared from 1 μg of total RNA by reverse transcription, using SuperScript® III First-Strand Synthesis System for RT-PCR (Life Technologies, Carlsbad, CA), following the manufacturer’s protocol.

### Cloning and subcloning of OBP3 genes

First strand cDNA (1 μL) was amplified in a G-STORM thermocycler in a total reaction solution (20 μL) containing 1 unit of *Taq* DNA polymerase (Life Technologies, Carlsbad, CA), 0.1 mM of each dNTP, 1.5 mM MgCl_2_, 0.2 μM of each PCR primer in the *Taq* Buffer supplied with the polymerase. The PCR primers were designed to match the *A. pisum* OBP3 sequence (GenBank accession: FM242529) using the online tool Primer3 (http://wwwgenome.wi.mit.edu/cgi-bin/primer) and excluding the signal peptide. The primers (Forward: 5′-AACATATGGAAAACAATCAACAAAACTCAAA-3′; Reverse: 5′-CTGAATTCTTACATGCTATTGCGTCTGAATT-3′) contained restriction sites (*Nhe*I and *Eco*RI) for cloning into the expression vector, the ATG codon (forward primer) and a stop codon (reverse primer) for the production of mature recombinant proteins with an additional methionine at position 1. After a first denaturation step at 94 °C for 3 min, 35 amplification cycles (30 sec at 94 °C, 30 sec at 55 °C, 45 sec at 72 °C) were done, followed by a final step of 10 min at 72 °C. The PCR product, with the expected size was purified from the agarose gel using QIAQuick Gel Extraction Kit (Qiagen, Hilden, Germany) according to the manufacturer’s instructions. The purified PCR product was then ligated into the pGEM T-Easy (Promega, Madison, WI) vector for 3 h at room temperature and then the product was used to transform *E. coli* Top10 chemically competent cells. The plasmid DNA was extracted from positive colonies using QIAprep Spin Miniprep Kit (Qiagen, Hilden, Germany) according to the manufacturer’s instructions and sequenced by Eurofins MWG (London, UK).

The pGEM/MvicOBP3 plasmid DNA containing the appropriate OBP sequence was digested with *Nhe*I and *Bam*HI restriction enzymes for 3 h at 37 °C and the digestion products were separated on a 1% agarose gel. The OBP insert was purified from the gel using QIAQuick Gel Extraction Kit (Qiagen, Hilden, Germany) according to the manufacturer’s instructions, and ligated into the expression vector pET17b (Novagen, Darmstadt, Germany), linearised with the same enzymes. The resulting pET17b/MvicOBP3 constructs were sequenced to confirm that they encoded the mature protein. Similarly, the bacterial expression construct pET17b/NribOBP3 for NribOBP3 was obtained by subcloning NribOBP3 genes in pGEM/NribOBP3 into pET17b vector but flanked with NdeI at 5′-end and EcoRI at 3′-end.

The computation of various physical and chemical parameters for the OBP3s was done using the online tool ProtParam on the Expasy SIB Bioinformatics Resource Portal (http://expasy.org/)^46^. Multiple sequence alignment of the OBP3s of *N. ribisnigri* and *M. viciae* was performed using ClustalW (http://www.ebi.ac.uk/Tools/msa/clustalw2/). The signal peptides were predicted by SignalP 4.1 (http://www.cbs.dtu.dk/services/SignalP/).

### Protein expression and purification of recombinant OBPs

For expression of recombinant proteins, each pET17b vector containing the MvicOBP3 sequence was used for transformation into BL21(DE3) pLysS *E. coli* cells. The protein expression was induced at A_600_ of 0.5–0.8 by addition of IPTG to a final concentration of 1 mM when the culture had reached a value of OD_600_ = 0.8. The cells were grown for a further 3 h at 37 °C, then harvested by centrifugation and sonicated. After centrifugation, the OBP was present as inclusion bodies and solubilisation was performed by denaturation in urea/DTT, renaturation and extensive dialysis against 20 mM Tris buffer pH 7.4, using a method described previously^19^. The protein was purified by anion-exchange chromatography as described previously^47^ on XK 26 column (Pharmacia, Uppsala, Sweden) filled with DE-52 resin (Whatman, Kent, UK), followed by gel filtration on Sephacryl S-200 HR (GE healthcare, Piscataway, NJ). The purified protein was stored at –20 °C in 20 mM Tris–HCl at pH 7.4. The gel filtration buffer is 20 mM Tris plus 150 mM NaCl at pH 7.4, and the storage buffer is 20 mM Tris pH 7.4. The protein was desalted prior crystallisation by dialysis with 20 mM Tris pH 7.4 buffer.

### Competitive fluorescence binding assays

Proteins were dissolved in 20 mM Tris-HCl buffer pH 7.4 and emission fluorescence spectra were recorded on a LS50 Luminescence Spectrometer (PerkinElmer, Buckinghamshire, UK) at 22 °C in a right angle configuration, with a 1 cm light path quartz cuvette and 5–10 nm slits for excitation and emission. To measure the affinity of the fluorescent probe NPN to the protein, a 2 μM solution of the protein in 20 mM Tris-HCl pH 7.4 was titrated with aliquots of 1 mM NPN in methanol to final concentrations from 1 to 14 μM. The probe was excited at 337 nm and emission spectra were recorded between 380 and 450 nm. The affinity of other ligands was measured in competitive binding assays, using NPN as the fluorescent reporter at 2 μM concentration titrated with each competitive ligand at between 0.1 and 15 μM concentrations. For determining binding constants, the intensity values corresponding to the maximum fluorescence emission were plotted against free ligand concentrations. We use GraphPad (http://www.graphpad.com/guides/prism/6/curve-fitting/index.htm?reg_one_site_competition_ki.htm) to determine the equilibrium dissociation constant Ki of ligand by measuring its competition for NPN and nonlinear regression curve fitting to the one site competitive binding model. This model fits the Ki of the ligand directly using the fixed concentration of NPN and the dissociation constant for NPN K_NPN_ obtained from a direct fluorescence titration and nonlinear regression curve fitting to one site binding model Y = Bmax*X/(K_NPN_ + X) + NS*X (X is the NPN concentration, Bmax is the maximum specific binding in the same units as Y, NS is the slope of nonspecific binding in Y units divided by X units). The corrected Ki is fitted directly rather than fitting an IC_50_ and modifying it with the Cheng and Prusoff equation^48^. The analysis assumes that the protein has one binding site and 100% active, and that the binding is reversible and at equilibrium. Tested compounds were EβF (Bedoukian Research, Danbury, CT, USA), β-pinene, (−)-α-pinene and (+)-limonene (Sigma, St. Louis, MO).

### Crystallisation and 3D structure solution

Purified and delipidated protein samples were concentrated by centrifugation for 2 minutes at 4 °C using a Vivaspin 20 with a 5,000 molecular weight cut off filter and an Eppendorf Centrifuge S415R set at 15600 rpm. An absorbance value at 280 nm was recorded using an ND-1000 Spectrophotometer and protein concentrations were calculated using the OBP primary sequence and Protein Calculator v3.3 (http://protcalc.sourceforge.net/). MvicOBP3 crystallised from a mother liquor of 0.3 *M* AmSO4, 30% (*w*/*v*) PEG 4000, 7.5% (*v*/*v*) Glycerol. NribOBP3 was crystallised from 20% PEG 6000, 0.1 M Sodium Citrate pH 5 10 mM ZnCl2. All crystals were harvested in 2 μL of mother liquor with glycerol adjusted to 30% (v/v). Crystal data were collected at Diamond Synchrotron, Harwell beamlines I24 and I04-1. Data was processed automatically through xia2^49^ to determine space groups and unit cell dimensions; in all cases the 3dii operation mode, which used XDS^50^ and scala^51^ and a wide scan of images for indexing, gave the best quality results. The structure of MvicOBP3 was solved with Phaser crystallographic software^52^ using sequential searches for two copies of three separate helices taken from the structure of the *Drosophila melanogaster* PBP LUSH (α-helices 2, 3 and 5). Phaser was run with MvicOBP3 resolution limited to 1.7 Å. This model was then run through SHELXE to improve phases by phase extension and density modification, including iterative model building and free lunch protocol^53^. The resulting map from SHELXE was autobuilt using Arp/wARP. The final Arp/wARP output was then run through 9 iterative stages of model refinement using COOT and RefMac5. Models were refined in COOT using difference map peak analysis, along with rotamer and density fit analysis. The final stage of refinement involved running Refmac5 with a resolution limit of 1.3 Å. NribOBP3 was solved using the MvicOBP3 structure as a search model in Phaser. All 121 residues, including 3 vector residues are visible in both chains of MvicOBP3. NribOBP3 has the last residue not modelled in 2 copies and the last two residues in one copy, but the vector residues are ordered.

### Binding site prediction by CASTp and blind docking approach

To identify putative binding pockets, the coordinate files for the monomers of MvicOBP3 and NribOBP3 were submitted to the Computed Atlas of Surface Topography of proteins (CASTp) server^37^. Residues, area and volume of the top-three pockets in the ranking were recorded. To corroborate the pocket prediction, a blind docking approach was done using Autodock Tools to set up the grid box for the OBP. Dimension and orientation for MvicOBP3 and NribOBP3 were 114 × 106 × 112 points (0.375 Å of spacing) and 7.992 (x-centre), −2.365 (y-centre) and 15.789 (z-centre). Dockings were run using Autodock Vina^39^ including the PDBQT files created for the protein and each ligand. Similar-structure interfaces were searched for comparison using the PISA interface search. Search results are ranked by a Q score; a structural alignment score from 0 to 1 that take into account alignment length and C-alpha RMSD.

### Refinement of Molecular docking

Binding affinities of MvicOBP3 and NribOBP3 were tested against (−)-α-pinene, β-pinene and EβF. The protein and ligand structures were prepared using Autodock Tools as PDBQT files. A grid box with dimension 38 × 38 × 38 points (0.375 Å spacing) and positioned in the centre of pocket A −0.056 (x-centre), 6.556 (y-centre) and 6.556 (z-centre) for MvicOBP3 was prepared. Similarly, dimension 38 × 38 × 38 points and coordinates 20.706 (x-centre), −21.413 (y-centre) and 1.748 (z-centre) were prepared for NribOBP3. Ligands were energy minimised and considered flexible while the protein was rigid. Autodock Vina was run through the command line in Windows. Every docked conformation and binding energy was recorded in a log file. Thus, binding energies were used to choose the best conformation and to calculate the theoretical inhibition constant (K_i_) with formula (1).


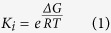


where ΔG is the binding energy, R is the gas constant and T is standard temperature. Docked ligands were visualised using PyMOL (http://www.pymol.org).

### Molecular dynamics

Simulations of conformational stability on both MvicOBP3 and NribOBP3 were performed using NAMD v2.9 and CHARMM36 force field. Proteins (chain A) were solvated with water (TIP3P model) in a cubic box with a minimum distance of 10 Å between the protein and the edge of the box. Likewise, the system was neutralised by adding Na^+^ or Cl^−^ randomly placed in the box. All protein preparation was carried out using Visual Molecular Dynamics software (VMD). Configuration files were prepared in order that the system was simulated under periodic boundary conditions with a cutoff radius of 12 Å for non-bonded interactions and a time step of 2 fs. Extensive energy minimisations (50000 steps) were performed followed by heating through short simulations of 1 ps at 50, 100, 150, 200, 250 and 300 K. Long simulations were kept at 300 K and 1 bar pressure in the NTP (referred to a constant number of particles, temperature and pressure) during 10 ns for each aphid OBP. Root-mean-square deviation (RMSD) trajectory tool in VMD was used to calculate the RMSD with reference to the starting structure. When the plotted RMSD did not showed any big changes, coordinates were analysed every 50 frames to obtain the best representative structure (lowest energy). Conformational changes were visually analysed by PyMOL software. Conformational energies (i.e. bond, angles, dihedral and improper features) for both MvicOBP3 and NribOBP3 were calculated using the DCD files resulted from MD simulations through NAMDEnergy plugin provided by VMD software, and plotted for the same software. A point mutation was performed on NribOBP3 structure using PyMOL, where Val98 was replaced by Ile (i.e. same residue as MvicOBP3) with the aim to assess overall conformational energy and the role of this main difference in sequence. After in silico mutation, 10000 steps of energy minimization were carried out by NAMD through VMD software. Conformational energy was calculated by NAMDEnergy plugin.

## Additional Information

**How to cite this article**: Northey, T. *et al*. Crystal Structures and Binding Dynamics of Odorant-Binding Protein 3 from two aphid species *Megoura viciae* and *Nasonovia ribisnigri. Sci. Rep.* **6**, 24739; doi: 10.1038/srep24739 (2016).

## Supplementary Material

Supplementary Information

## Figures and Tables

**Table 1:** Comparison between MvicOBP3 and NribOBP3.

**GESAMT**	**NribOBP3**			**MvicOBP3**	
	**A**	**B**	**D**	**A**	**B**
NribOBP3 A	0.00/114	0.32/111	0.53/112	0.98/110	0.82/109
NribOBP3 B		0.00/112	0.52/110	0.94/108	0.8/108
NribOBP3 D			0.00/114	0.94/111	0.77/109
MvicOBP3 A				0.00/115	0.59/109
MvicOBP3 B					0.00/114

Calpha RMSD (Å) between chains of the aphid OBP3s using GESAMT in

CCP4MG^54^. The second number refers to the number of matching residues.

**Table 2:** *In silico* binding energies and K_i_ for MvicOBP3 and NribOBP3.

**Molecule**	**MvicOBP3**		**NribOBP3**	
	**Binding energy(kcal mol**^**−1**^)	**K**_**i**_**(μM)**	**Binding energy(kcal mol**^**−1**^)	**K**_**i**_**(μM)**
NPN	−7.3	4.4	−7.4	3.7
(−)-α-pinene	−5.1	181	−5.1	181
β-pinene	−5.2	153	−5.2	153
(+)-limonene	−5.4	109	−4.3	699
(*E*)-β-farnesene	−5.5	92	−5.1	181
