# Supplementary material for: Crystal Structures and Binding Dynamics of Odorant-Binding Protein 3 from two aphid species Megoura viciae and Nasonovia ribisnigri
Source: Sci Rep. 2016 Apr 22;6:24739. doi: 10.1038/srep24739 (PMC4840437; doi:10.1038/srep24739)

Crystal Structures and Binding Dynamics of Odorant-Binding Protein 3 from two aphid species *Megoura viciae* and *Nasonovia ribisnigri*

Tom Northey[¶,2,5], Herbert Venthur[¶,1,4], Filomena De Biasio[¶,1,3], Francois-Xavier Chauviac[2,6], Ambrose Cole[2], Karlos Antonio Lisboa Ribeiro Junior[1], Gerarda Grossi[3], Patrizia Falabella[3], Linda M. Field[1], Nicholas H. Keep[2], Jing-Jiang Zhou[*,1]

Supplementary Table 1. Data collection and refinement parameters for apo-MvicOBP3 and NribOBP3 structures.

Crystal	*M. viciae*	*N. ribisnigri*
PDB CODE	4Z39	4Z45
**Data collection**		
Wavelength (Å)	0.9686	0.9173
Space group	P1 (1)	$P3_121$ (152)
Cell dimensions		
a, *b*, *c* (Å)	36.2, 41.4, 44.3	87.3,87.3,95.1
α, β, γ (^{o})	100.3, 101.7, 105.0	90, 90, 120
Resolution (Å)[a]	38.8-1.30	59.6-2.02
	(1.32-1.30)	(2.07-2.02)
Total number of reflections [a]	157442 (6151)	558861 (41849)
Number of unique reflections [a]	52318 (2327)	27946 (2051)
R_{merge} [a]	0.089 (0.398)	0.149 (0.738)
R_{meas} [a]	0.125 (0.563)	0.156 (0.776)
R_{pim} [a]	0.089 (0.398)	0.035 (0.172)
CC (1/2) [a]	0.976 (0.716)	0.998 (0.938)
Solvent content (%)	45.1	51.9
Molecule/asymmetric unit	2	3
Wilson B-factor (Å^2)	17.5	8.8
I/σI [a]	9.6 (1.9)	16.3 (4.6)
Completeness (%)[a]	90.1 (81.7)	100 (100)
Redundancy [a]	3.0 (2.6)	20.0 (20.4)
**Refinement**		
Resolution (Å)[a]	38.8-1.30	69.2-2.02
	(1.33-1.30)	(2.07-2.02)
Reflection, working	49450	26520
Reflection, free	3375	1946
R_{work}/R_{free} (%)	12.5/16.1	16.4/20.7
No of non-H atoms	2115	3133
Protein	A,969 B,977	A,940 B,943 D, 941
Others	12 (Glycerol)	
	20 (Sulphate)	

Water	199	366
B factors (Å^2)[b]	23.8	26.6
Protein	A, 22.7 B, 23.6	A,19.5 B,25.6 D,30.2
Others	40.5 (Glycerol)	
	58.0 (Sulphate)	
Water	36.0	31.5
Rmsds		
Bond lengths (Å)	0.034	0.026
Bond angles (°)	2.8	2.1
Ramachandran plot		
Favoured (%)	97.8	98.2
Allowed (%)	1.8	1.8
Outliers (%)	0.4	0

[a] Values in parentheses are for the highest-resolution shell. [b] Average over all atoms.

$$R_{merge} = \frac{\sum_{hkl}\sum_j \left|I_{hkl,j} - \langle I_{hkl}\rangle\right|}{\sum_{hkl}\sum_j I_{hkl,j}}$$

$$R_{means} = \frac{\sum_{hkl}\sqrt{\frac{n}{n-1}}\sum_{j=1}^{n}\left|I_{hkl,j} - \langle I_{hkl}\rangle\right|}{\sum_{hkl}\sum_j I_{hkl,j}}$$

$$R_{p.i.m} = \frac{\sum_{hkl}\sqrt{\frac{1}{n-1}}\sum_{j=1}^{n}\left|I_{hkl,j} - \langle I_{hkl}\rangle\right|}{\sum_{hkl}\sum_j I_{hkl,j}}$$

where I_{hkl} is the reflection intensity and $<I_{hkl}>$ is the average intensity for multiple measurements of that reflection.

Supplementary Table 2. Comparison of MvicOBP3 and NribOBP3 with other insect OBPs by DALI and PDBeFold search.
Top 4 distinct PDB entries and lowest 2 distinct PDB entries above first non-IPR00137 protein shown.

RANK MvicOBP3 DALI	Chain	Z-score	RMSD	%id	nres	Form	Macromolecule	Molecule in Asymmetric Unit (ASU)	Ligand Binding	Stoichiometry
1	4F7F-D	9.7	3.4	12	107	complex	*A.gambiae* OBP20	4	ligand in pocket	A2
3	3V2L-A	9.4	3.6	13	107	complex	*A.gambiae* OBP20			
6	3VB1-A	9.0	3.7	12	105	apo	*A.gambiae* OBP20			
7	3R72-A	8.9	3.4	13	99	complex	*A.mellifera* OBP5	1		monomer
|	|	|	|	|	|	|	|	|		|
166	1C3Z-A	4.4	5.1	17	78	complex	*T.Mollitor* 14.3k Haemolymph protein	1		monomer
167	2FJY-A	4.4	3.8	11	92	apo	*B.mori* PBP (Cterm in core)	2		monomer

RANK NribOBP3 DALI	Chain	Z-score	RMSD	%id	nres	Form	Macromolecule	Molecule in Asymmetric Unit (ASU)	Ligand Binding	Stoichiometry
1	4F7F-D	9.2	3.2	14	103	complex	*A.gambiae* OBP20	4	ligand in pocket	A2
2	1ORG-B	9.1	3.4	18	106	complex	Rhyparobia maderae OBP	2		monomer
4	3V2L-A	9.1	3.4	14	103	complex	*A.gambiae* OBP20			
6	3R72-A	8.9	3.4	15	101	complex	*A.mellifera* OBP5	1		monomer
|	|	|	|	|	|	|	|	|		|
158	3R1P-D	5.0	3.8	20	80	apo	*A.gambiae* OBP7	6		monomer
160	3R1O-B	4.8	4.3	19	83	apo	*A.gambiae* OBP7	6		monomer

RANK MvicOBP3 PDBeFold	Chain	Z-score	RMSD	%id	nres	Form	Macromolecule	Molecule in Asymmetric Unit (ASU)	Ligand Binding	Stoichiometry
1	4PT1-B	4.8	2.3	14	83	complex	*L. migratoria* OPB1	2		monomer
2	3R72-A	4.8	2.8	14	94	complex	*A.mellifera* OBP5	1		monomer
3	3D78-A	4.2	2.7	17	81	complex	*A.mellifera* PBP1	2		homo 2-mer - A2
4	4F7F-D	4.1	2.7	14	96	complex	*A.gambiae* OBP20	4		A2
|	|	|	|	|	|	|	|	|		|
85	3D75-A	2.4	3.1	12	81	complex	*A.mellifera* ASP1	2		homo 2-mer - A2
86	3R1P-A	2.3	3.1	14	74	apo	*A.gambiae* OBP7	6		monomer

RANK NribOBP3 PDBeFOLD	Chain	Z-score	RMSD	%id	nres	Form	Macromolecule	Molecule in Asymmetric Unit (ASU)	Ligand Binding	Stoichiometry
1	4PT1-B	4.8	2.2	17	81	complex	*L. migratoria* OBP1	2		monomer
2	3R72-A	4.6	2.9	14	98	complex	*A.mellifera* OBP5	1		monomer
3	3VB1-A	4.5	2.7	13	89	complex	*A.gambiae* OBP20	4		monomer
4	2H8V-A	4.3	2.2	18	71	complex	*A.mellifera* ASP1	1		monomer
|	|	|	|	|	|		|	|		|
70	3B86-A	2.6	3.1	16	101	complex	*D. melanogaster* LUSH	2		monomer
71	2L2C-A	2.1	3.5	9	88	apo	*C.quinquefasciatus* OBP1 (NMR)	1		monomer

Matches to the same PDB have been omitted, but they are not always consecutive. Eg The 4 chains of 4F7F match MVicOBP3 in PDBEfold at ranks 4,13,19 and 20.

Supplementary Figure 1. OBP3 peptide sequence alignment between MvicOBP3, NribOBP3 and ApisOBP3. The residues that form the proposed binding site are highlighted in orange (MvicOBP3) and blue (NribOBP3), and Tyr30 is indicated by a red arrow.

```
                                           ↓
MvicOBP3    RFTTEQIDYYGKACNASEDDLVVVKSYKVPSSETGKCLMKCMITKLGLLNDDGSYNKTGM
NribOBP3    RFTTEQIDYYGKACNASEDDLVVVKSYKVPSSETGKCLMKCMITKLGLLNDDGSYNKTGM
ApisOBP3    RFTTEQIDYYGKACNASEDDLVVVKSYKVPTTETGKCLMKCMITKLGLLNDDGSYNKTGM
            ******************************::****************************

MvicOBP3    EAGLKKYWSEWSTEKIESINNKCYEEALLVSKEVIATCNYSYTVMACLNKQLDLDKST
NribOBP3    EAGLKKYWSEWSTEKIESINNKCYEEALLVSKEVVATCNYSYTVMACLNKQLDL----
ApisOBP3    EAGLKKYWSEWSTEKIESINNKCYEEALLVSKEVVATCNYSYTVMACLNKQLDLDKST
            **********************************:*******************::::
```

Supplementary Figure 2. Expression and purification of MvicOBP3 and NribOBP3. Electrophoretic analysis (SDS-PAGE) of crude bacterial pellets before (UnIN) and after (IN) induction with IPTG for MvicOBP3 (lane 2 and 3) and for NribOBP3 (lane 4), and of purified samples (P) of the proteins. Molecular weights of markers (M) are from the top, 66, 45, 29, 20 and 14 kDa.

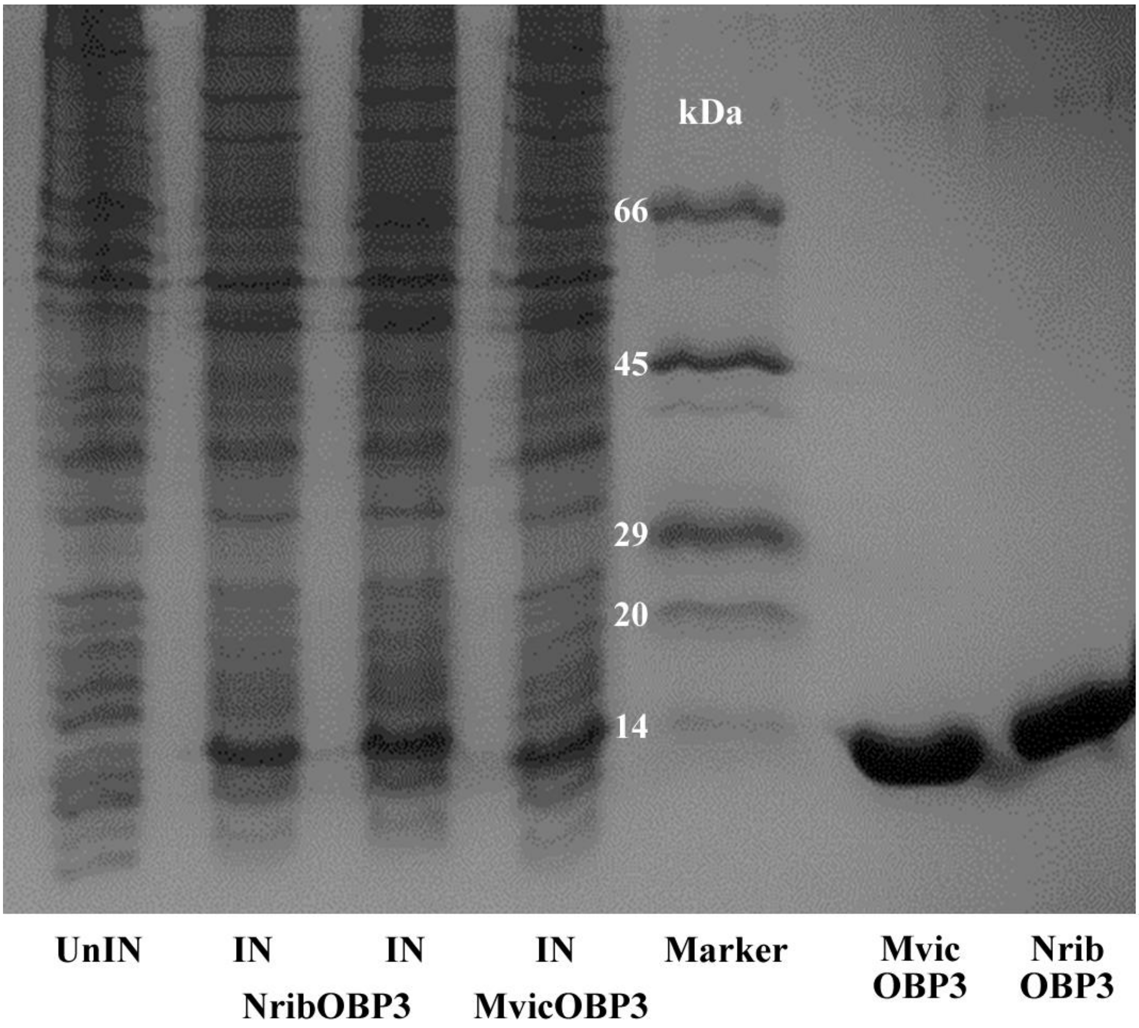

Supplementary Figure 3. Superimposed structures for MvicOBP3 (A) and NribOBP3 (B) before (green) and after (light blue) 10 nsec simulation of molecular dynamic analysis.

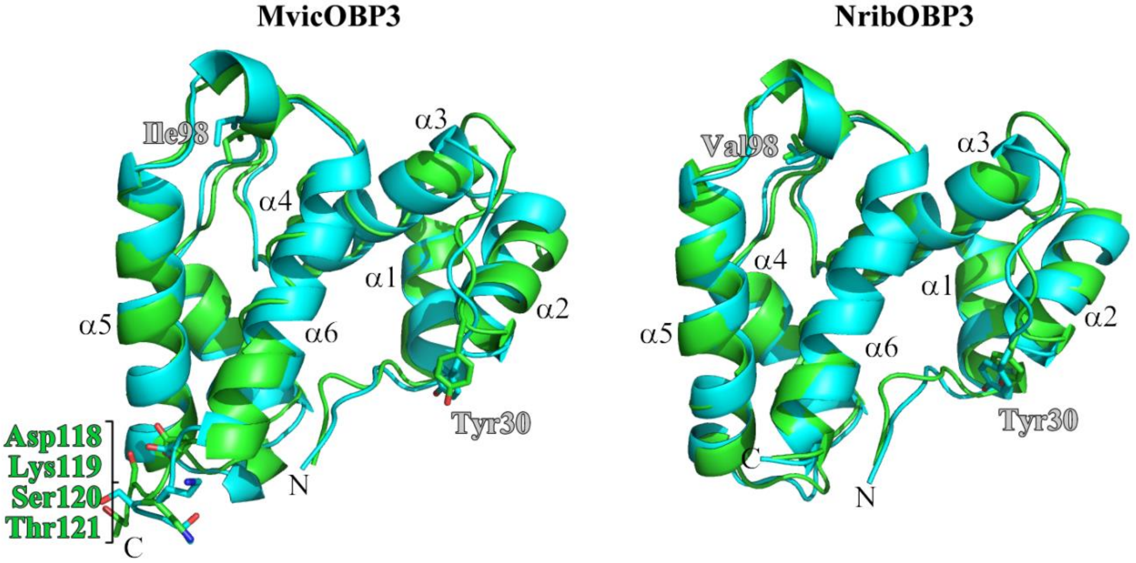

Supplementary Figure 4. A close look at the binding region of MvicOBP3 (top right box) and NribOBP3 (bottom right box) before (green for MvicOBP3 and magenta for NribOBP3) and after (light blue for MvicOBP3 and yellow for NribOBP3) 10 nsec simulation of molecular dynamic analysis. C and N indicate C- and N-terminals, respectively. Alphas 1 to 6 indicate the number and position of ribbons for all the 3D structures.

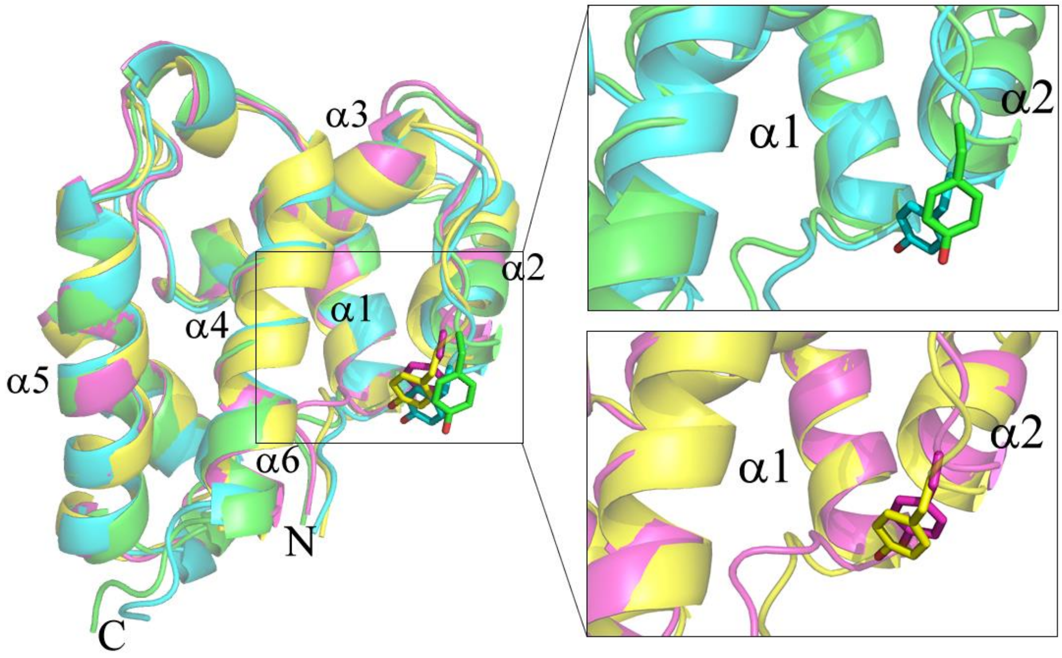

Supplementary Figure 5. Binding of recombinant MvicOBP3s (left) and NribOBP3 (right) to NPN (top panel) and the alarm pheromone components (bottom panel). A 2 µM solution of the protein in 20 mM Tris pH7.4 was titrated with 1 mM solution of NPN or each of pheromone components in methanol to final concentrations of 0.1-20 µM.

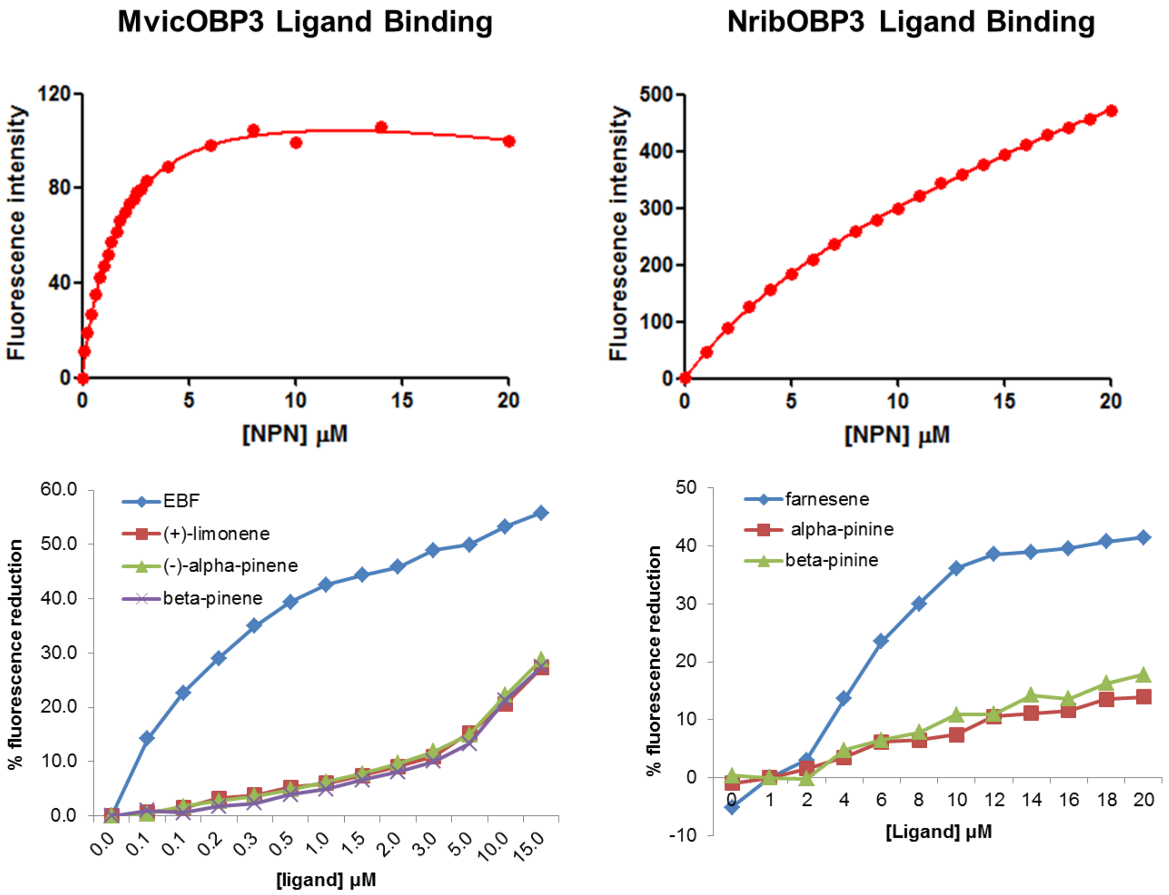

Supplement: Supplementary Information [file srep24739-s1.pdf]
